# Central [CNS] and Peripheral [Gastric Tissue] Selective Monitoring of Somatostatin (SRIF) with Micro-Sensor and Voltammetry in Rats: Influence of Growth Factors (GH, EGF)

**DOI:** 10.3390/bios7040053

**Published:** 2017-11-17

**Authors:** Francesco Crespi

**Affiliations:** Biology, GSK, via Fleming 4, 37135 Verona, Italy; fm.crespi@libero.it

**Keywords:** somatostatin (SRIF), rat, CNS, stomach, differential pulse voltammetry (DPV) carbon fiber micro-electrode (µCFE), growth factors

## Abstract

Somatostatin (SRIF) is widely distributed throughout the body, and regulates the endocrine system via interactions with various hormones, including the pituitary growth hormone, the thyroid stimulating hormone and the majority of the hormones of the gastrointestinal tract. SRIF is present in the central nervous system (CNS), where it affects rates of neurotransmission, and is also reported to be active in the intestinal tract, with evidence that stressed rats present a significant decrease in antral somatostatin-like immunoreactivity (SLI). Analysis of SRIF has mainly been carried out by means of radioimmunoassay methods. Here, we propose the use of an electrochemical method, such as voltammetry, applied with carbon-based sensors and, in particular, the combination of differential pulse voltammetry with treated carbon fiber micro electrodes (DPV-µCFE) to facilitate the analysis of such peptidergic electro active hormones in the rat striatum and gastric tissue; the effect of growth hormone (GH) and epidermal growth factor (EGF), in particular, upon the SRIF signal has been studied in such tissues.

## 1. Introduction

The somatotropin release-inhibiting factor (SRIF) Somatostatin is a hormone distributed throughout the body. It is central in the regulation of the endocrine system via its interactions with pituitary growth hormone, thyroid stimulating hormone, and most hormones of the gastrointestinal tract (for a review, see [1]). Furthermore, it interacts with G protein-coupled SRIF receptors thus affecting neurotransmission and cell proliferation [2]. SRIF is also recognized as a growth hormone-inhibiting hormone (GHIH).

It is known that SRIF influences the proliferation of both normal and tumorigenic cells [3,4]. However, in a number of cancerous cell lines, SRIF has also been found to inhibit EGF-induced cell proliferation [5,6].

Prolonged infusion of SRIF has been found to inhibit gastric mucosal cell division [7]. In addition, administration of somatostatin together with gastrin, has been shown to diminish the gastrin-mediated stimulation of cell proliferation in the gastric mucosa, indicating an interaction also between the two hormones [8,9].

It has been shown that, in rats submitted to stress, e.g., by water immersion, the ulcer index of gastric mucosa is significantly higher than that in control rats [10,11]. In particular, the stressed rats presented a significant decrease in antral somatostatin-like immunoreactivity (SLI). It is also known that treatment of stressed rats with epidermal growth factor (EGF), results in an ulcer index significantly lower than that in stressed rats treated with vehicle. This indicates that EGF exerts a cyto-protective activity on gastric mucosa [12,13] and, taken together with the evidence that EGF treatment determines levels of antral SLI significantly higher than that in control rats, proposes a role for EGF in preventing stress ulcer formation. Furthermore, it suggests an involvement of the endogenous SLI in its anti-ulcer function.

Analysis of SRIF has been carried out mainly by means of radioimmunoassay methods [12,14], i.e., measured in plasma by RIA after ethanol extraction [15] or by RIA-[125I] LTT SRIF-28 binding [16].

Here, we apply an electrochemical method, i.e., differential pulse voltammetry (DPV), in order to analyze the feasibility of monitoring SRIF in rat gastric preparation. Up until now, the combination DPV-µCFE has permitted the development of accurate tests for endogenous chemicals in discrete brain regions of rodents [17]. In particular, amino acids [AAs] such as tyrosine, tryptophan, and cysteine, as well as neuropeptides that these AAs in their structure, show electroactivity when analyzed with DPV-µCFE, with the oxidative potential reaching between +600 and +900 mV in vitro, i.e., in PBS buffer solution at pH 7.4. In particular, we have observed that, in vivo in rat striatum, the signal was monitored at approx +800 mV, and Peak 5 corresponded to the oxidation of SRIF. Indeed, the in vitro oxidation of SRIF, as well as that of structurally related peptides at such potential, has previously been established [18].

## 2. Methods & Results

### 2.1. DPV and Micro-Sensors

Voltammetry, and differential pulse voltammetry (DPV) in particular, is an electrochemical technique that, used in association with specifically treated carbon fiber micro electrodes (µCFE), allows the detection of catecholamines, serotonin and peptides simultaneously in discrete brain regions of anesthetized or conscious freely moving rats [19].

This methodology complies with the majority of the conditions required for examining specific compounds in the extracellular fluid (for a review see [20]); briefly:

- The undersized dimensions of the probe allow measurements with minimal damage to the nervous tissue and disturbance to the animal.
- The area sampled is approximately 10^–^6 mm^3^: this means high anatomical resolution of the location of measurement within discrete brain regions.
- Fast, continuous measurements in vivo, in situ in real time, without requiring perfusion or sample preparation or chromatographic separation or radiolabelled transmitter supplies.
- Feasibility of performing DPV in freely moving rodents; this solves the problems associated with anesthetics permitting correlations within neuronal activities.
- Wireless DPV measurements allow electrochemical studies in completely free-moving situations [21].

### 2.2. In Vitro Studies

By means of untreated carbon-paste electrodes it has been revealed that various amino acids and neuropeptides are electro active [22]. Therefore, they have been tested here with DPV-µCFE, and the results are presented in Table 1. In particular, it appears that CCK-8, SRIF and alpha-MSH oxidize in vitro at approximately +800 mV.

Briefly, the electrochemical activity of such compounds dissolved in saline (vehicle, NaCl 0.9%) was determined in vitro by the association DPV-µCFE performed as described previously [17,20] in a 500 µL 1 mM solution of each peptide.

**Figure 1:**
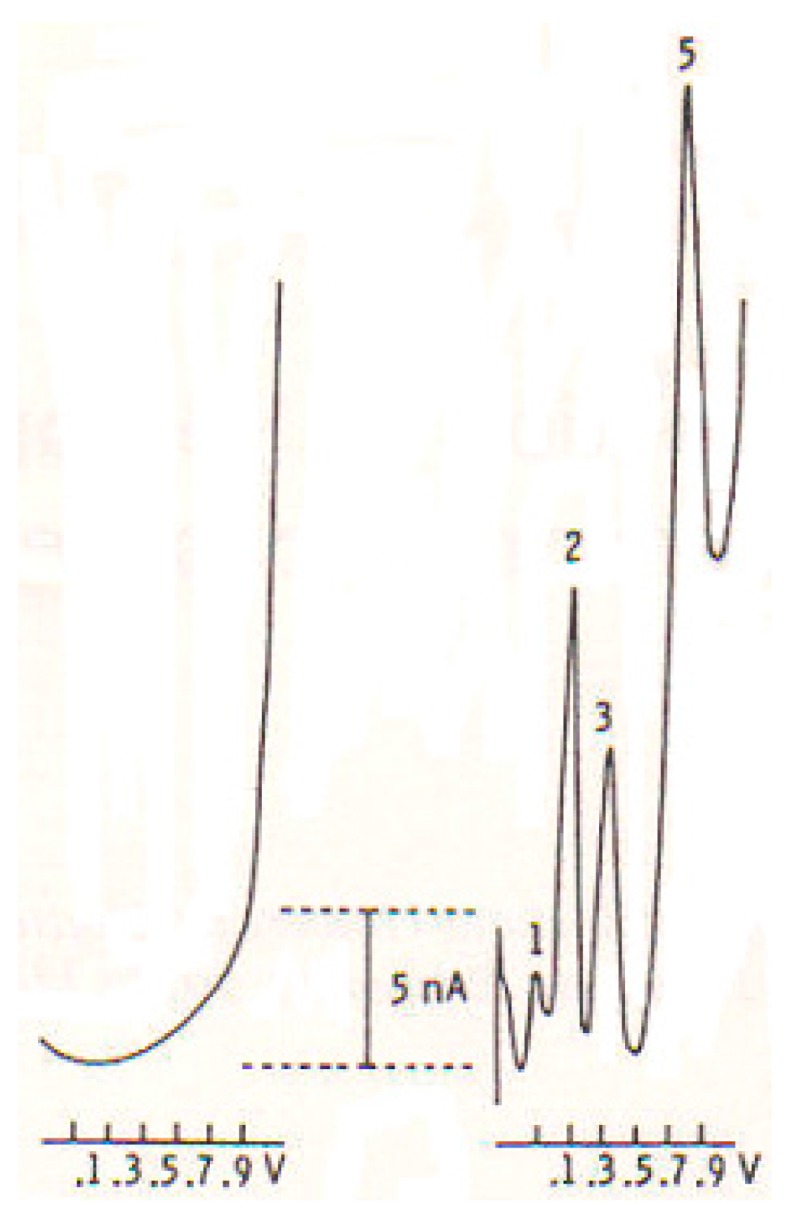
In vitro DPV-µCFE scans obtained in PBS, 0.1 M; pH 7.4 (LEFT) or in a PBS solution containing a mixture of ascorbic acid (AA) 5 mM; DOPAC, 50 µM; 5HIAA, 25 µM and SRIF 1 mM; in PBS, 0.1 M; pH 7.4. Peak 1: AA at −50 mV; Peak 2: DOPAC at +100 mV; Peak 3: 5HIAA at +300 mV; and Peak 5: SRIF at +800 mV (modified from [18]).

The µCFE were prepared using a 12 µm-diameter carbon fiber (Carbone Lorraine, Lyon, France) and were electrically treated firstly with a voltage from zero to 3 Volts, 70 Hz, 10 s, then with continuous potentials (+1.5 Volts, 5 s and −0.9 Volts, 5 s), so as to permit the measurement of three oxidation signals related to ascorbic acid, dopamine and serotonin metabolites, respectively, as well as that of a further oxidation peak when the DPV recordings were made in the same solution with the addition of the amino acids or neuropeptides cited above (see Figure 1).

### 2.3. In Vivo CNS Studies

**Figure 2:**
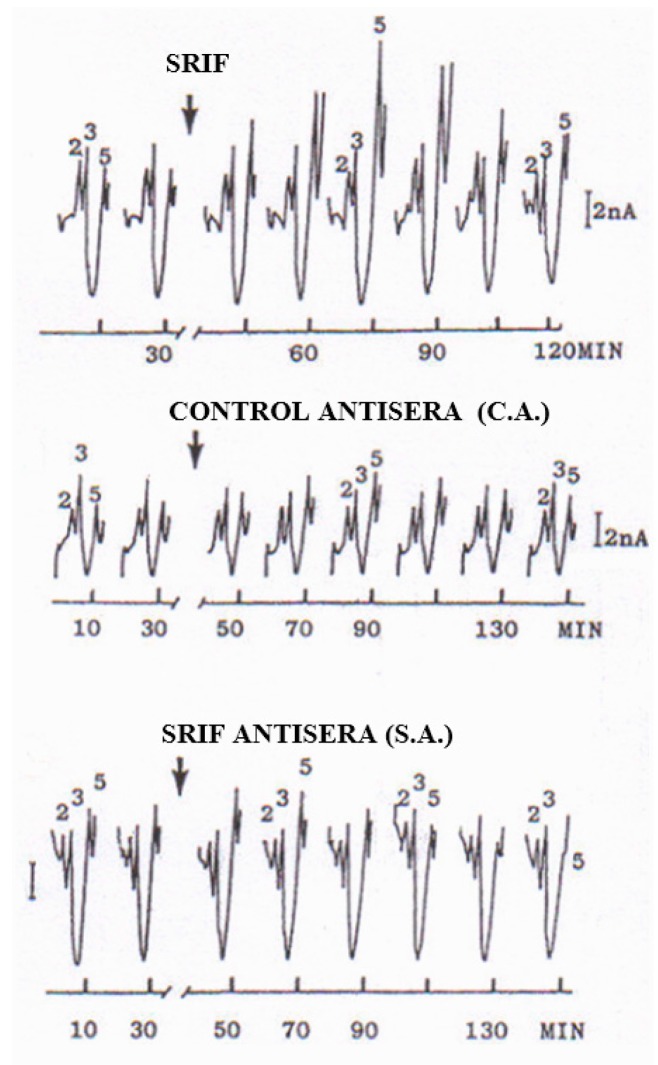
Typical DPVoltammograms monitored in the striatum of anesthetized rats following local injection of SRIF (top, n = 1), SRIF antisera (S.A.), i.e., antibodies for SRIF (rabbit polyclonal, IgG antiSRIF AB5494); Millipore (MERCK) (middle, n = 1); or control antisera (C.A.), i.e., non specific antibodies in the striatum of a single animal (bottom, n = 1). See Table 2 for data obtained in groups of rats treated as above (n = 7 each treatment), as well as with NaCl 0.9% (control group, n = 9), Bacitracin (n = 5), GH (n = 5) and Cysteamine (n = 5).

In vivo, in the rat striatum prepared for DPVoltammetric studies as previously described [19,20], local injection of 2 µg/µL of these peptides (ß-endorphin, CCK-8, SRIF or alpha-MSH) showed that SRIF is the one producing the largest increase of Peak 5 when compared to the other peptides, i.e., to approx. +475% of control versus +150% of control, respectively (see Figure 2).

Various treatments were successively performed in other groups of rats; in particular, Table 2 shows the results, presented as % of control values, obtained following treatment with:

- Bacitracin, which strongly inhibits peptidase activity, as described in [23], resulted in a large increase of Peak 5, therefore supporting it as a peptidergic signal.
- Cysteamine, which is a selective depletory of cerebral SRIF [24], was followed by a rapid decrease until disappearance of Peak 5.
- SRIF antisera, i.e., antibodies for SRIF (rabbit polyclonal, IgG antiSRIF AB5494); Millipore (MERCK), but not control antisera, i.e., non-specific antibodies, as described by Funato, et al. [25], resulted in a rapid decrease until disappearance of striatal Peak 5.
- GH 2 µg in striatum determined a transitory but significant increase of Peak 5, thus supporting the assumption that GH may regulate its own central levels by increasing endogenous SRIF [26], which is known to act as an inhibitor of GH-releasing factor [27].

### 2.4. Ex Vivo Gastric Tissue Studies

A group of adult male rats (220 g weight) was selected, and the stomach antrum was obtained as described [11]. Briefly each rat was anesthetized, and then sacrificed so as to extract the stomach, which was distended with 10 mL of cold saline for the purpose of stretching and fixing the mucosa. Then, each stomach was dissected, with the larger curvature and the antrum subsequently being divided off from the oxyntic gland area. Each antrum was then divided, and each part was incubated during 120 min with:

(1) Vehicle (PBS), or
(2) Antibodies for SRIF (rabbit polyclonal, IgG antiSRIF AB5494); Millipore (MERCK S.p.A., Vimodrone, Milan, Italy) or with non-specific antibodies as described by Funato, et al. [25], or
(3) Cysteamine 1 mM, or
(4) Epidermal growth factor (EGF) 1 mM.

**Figure 3:**
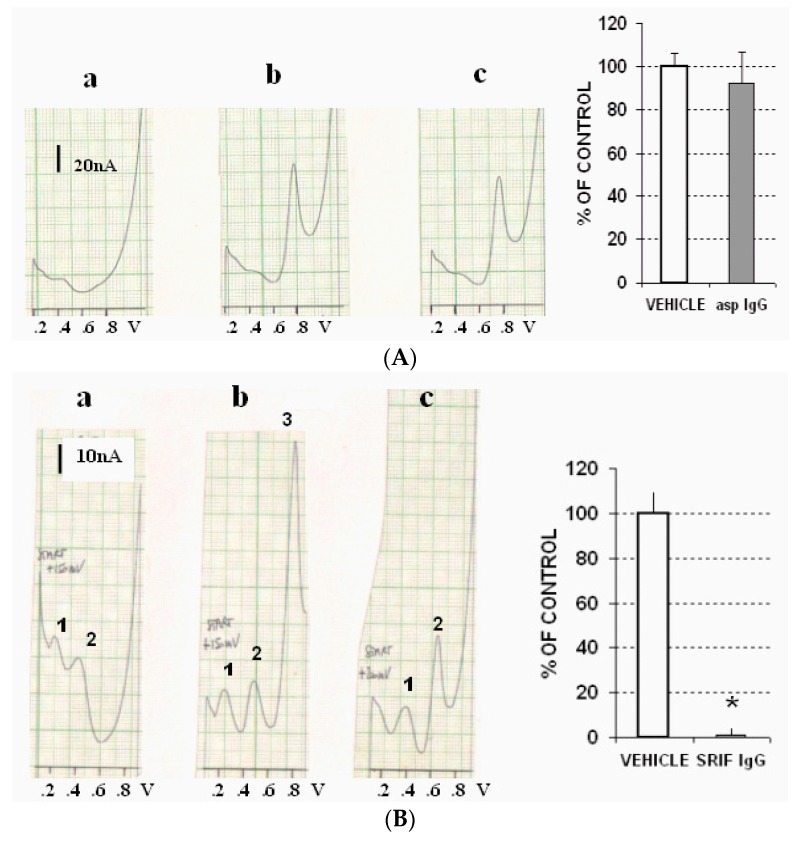

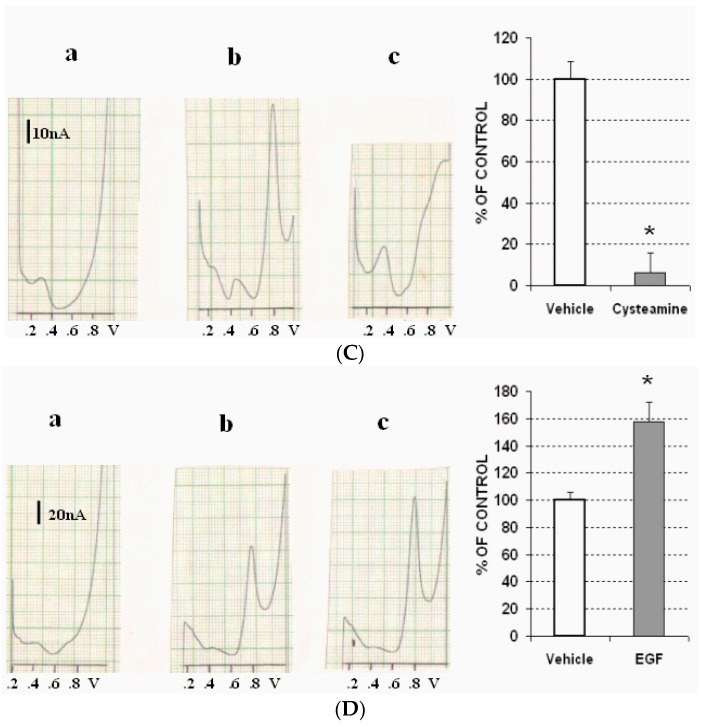
(**A**) Left: DPV-µCFE scans obtained in (**a**) vehicle (PBS); (**b**) in the gastric tissue of a single animal incubated with vehicle; (**c**) in the gastric tissue of a single animal incubated with aspecific IgG antisera (asp IgG). Note that the oxidation signal monitored at approximately .8 V (i.e., 800 mV) and measuring approximately 60 nanoAmperes (nA) superimposes the signal detected in the gastric tissue incubated with vehicle. Right, data obtained in antral preparation from 5 rats, incubated with vehicle or aspecific IgG antisera [asp IgG]; (**B**) Left: DPV-µCFE scans obtained in (**a**) vehicle (PBS); (**b**) in the gastric tissue of a single animal incubated with vehicle; (**c**) in the gastric tissue of a single animal incubated with specific IgG antisera (SRIF IgG); note that the peak monitored at +800 mV (denoted as peak 3 in this figure) and having a size of approximately 60 nA is no longer detected following incubation with specific IgG antiSRIF [peaks 1 and 2 are detected at lower oxidation potential, i.e., +200 or +400 mV, respectively, and are not affected by SRIF IgG]. Right, data obtained in antral preparation from 5 rats, incubated with vehicle or specific IgG antisera; (**C**) Left: DPV-µCFE scans obtained in (**a**) vehicle (PBS); (**b**) in the gastric tissue of a single animal incubated with vehicle; (**c**) in the gastric tissue of a single animal incubated with cysteamine; note that the peak monitored at +800 mV is greatly decreased. Right, data obtained in antral preparation from 5 rats, incubated with vehicle or cysteamine; (**D**) Left: DPV-µCFE scans obtained in (**a**) vehicle (PBS); (**b**) in the gastric tissue of a single animal incubated with vehicle; (**c**) in the gastric tissue of a single animal incubated with EGF: note that the peak monitored at +800 mV is significantly increased, i.e., from approximately 60 nA to approximately 85 nA.

Successively, each gastric tissue was homogenized in PBS at zero degrees (°C) in a ratio of 1:4 weight/volume using a glass-glass homogenizer potter (SAVI, Milan, Italy). All the homogenates were centrifuged at 11,000× *g* at 4 °C for 10 min. Then, each clear supernatant was collected, and DPV-µCFE measurements were performed for the one obtained from the antrum fraction incubated in vehicle, showing the presence of 2 oxidation signals: a small oxidation signal at approximately 400/450 mV, and a taller oxidation signal at approximately 800 mV. Neither signal was significantly affected by incubation in aspecific IgG (Figure 3A). In contrast, incubation in specific SRIF antisera resulted in a significant drop in the size of the peak recorded at 800 mV (Figure 3B). Furthermore, this signal almost vanished following incubation in cysteamine (Figure 3C). In contrast, incubation in EGF resulted in significant selective increase of the size of the peak monitored at 800 mV (Figure 3D).

## 3. Statistical Analysis

Row data were subjected to ANOVA, with comparison between “control” (vehicle) and “treatments” values performed using the Tukey test. Then, the results were presented as % of control values, mean ± SD, * *p* < 0.05.

## 4. Discussion and Conclusions

SRIF is highly involved in the regulation of various tasks of the endocrine and nervous system, where it binds to selective receptors on the cell surface, thereby producing its biologic action [28]. Presence of SRIF has also been detected in the salivary glands, in the thyroid and in the gastrointestinal tract (for reviews, see refs. [1,2]. Moreover, large distribution of SRIF within the CNS has been demonstrated, and quite high amounts of SRIF are present in the striatum [29,30,31]. Indeed, local injection into striatum of the effective peptidase inhibitor Bacitracin resulted in a large increase of Peak 5, therefore supporting it as a peptidergic signal. Similarly, local injection of SRIF produces the largest increase of striatal peak 5 when compared to the other peptides with similar oxidation potentials.

It has been reported that cerebral SRIF and prolactin are selectively affected by the thiol reagent Cysteamine [14,32,33]. In an additional ex vivo investigation, a selective decrease of SRIF in the rat CNS following systemic treatment with cysteamine has been observed [34]. Accordingly, a reduction of levels of SRIF has been reported following local treatment of slices of rat striatum with cysteamine [35]. Similarly, Kwok et al. [36] detected a prompt fall in immunoreactive SRIF (IR-SRIF) levels when measuring in the hypothalamus of rat brain.

In agreement with those in vitro and ex vivo studies, for in vivo voltammetric measurements, it was observed that the size of the oxidation peak monitored in the rat striatum at approximately +800 mV and so-called Peak 5 were diminished following systemic cysteamine.

Again, while intra-striatal treatment with control aspecific antisera had no significant effect on the size of Peak 5, purified SRIF antisera injected locally into the rat striatum of anesthetized rats caused the eventual disappearance of this voltammetric signal within 120 min. This effect was probably due to the combination of the specific antisera with SRIF in the extracellular space, resulting in the prevention of SRIF oxidation at the surface of the working electrode.

This in vivo voltammetric data is therefore in accord with the estimation that this peak is due to (a) a peptide, and (b) possibly SRIF, which could then be the main constituent of this DPV-µCFE signal in the striatum of the rat brain

Central and peripheral relationships between SRIF and growth hormone (GH) have been described [37,38], and it has been proposed that central levels of GH are controlled via a feedback mechanism linked to GH-stimulated production and release of SRIF [26,39].

Here, local injection of GH in striatum resulted in a short but significant increase of the size of Peak 5, thus further supporting the chemical nature of this DPVoltammetric signal, as well as the hypothesis that GH may regulate its own central levels via increasing endogenous SRIF, which is known to act as an inhibitor of GH-releasing factor [27,40]. 

Somatostatin-like immunoreactivity (SRIF-LI) has also been measured in the rat stomach, and it was observed that the addition of exogenous norepinephrine and dopamine considerably increased the secretion of gastric SRIF in a dose-dependent fashion [41,42,43]. Therefore, previous DPVoltammetric observations of increased levels of striatal Peak 5 following treatment with apomorphine [44] further support the chemical nature of such signal in vivo.

In the stomach lumen cysteamine is active at the cellular level, and it has been observed that it is responsible for the decay of the present SRIF and/or for the severe decline of SRIF synthesis [14,45]. Accordingly, the voltammetric data obtained here in the stomach antrum indicated the clear influence of incubation with cysteamine, which resulted in a large reduction of the putative gastric SRIF signal recorded at 800 mV. Similarly, incubation with specific antisera depleted this signal in gastric preparation, just as it was described to happen in the rat striatum.

In other studies, in rats submitted to stress applied by water immersion, a significant decrease in antral SRIF like immunoreactivity (SLI) was monitored, along with evidence of stress-induced antral ulceration. On the other hand, a considerable increase in antral content of SLI was obtained via injection of pentagastrin and/or EGF [12]. This is in agreement with our experiments, which show that antral incubation with EGF resulted in a significant increase of the electrochemical signal occurring at +800 mV in the rat gastric tissue when using ex vivo DPV-µCFE. Thus, this data supports the direct influence of EGF upon the DPV-µCFE antral signal recorded at +800 mV, and provides support for the assumption that this voltammetric peak is linked to gastric SRIF oxidation. Furthermore, this data is parallel to an earlier voltammetric in vivo observation of direct relationship between cerebral SRIF and Somatomedin C [44], which is known to interact with other growth factors, such as growth hormone (GH), both peripherally and centrally [46,47].

Altogether, the presented voltammetric data support the literature description of the influence of cysteamine, EGF, specific SRIF antisera upon SRIF, and confirm the chemical nature of both the striatal and the gastric signal recorded at 800 mV with DPV-µCFE as corresponding to oxidation of central and peripheral (gastric) SRIF. 

Up until now, analysis of SRIF has mainly been carried out by means of radioimmunoassay methods [12,14], i.e., measured in plasma by RIA after ethanol extraction [15]. The electrochemical method DPV-µCFE proposed here for the detection of SRIF presents various advantages over methods based on the preparation of samples and/or separation steps, as it allows rapid, direct, concomitant detection of different chemicals based upon specific oxidative (or red-ox) potentials in either in vitro, ex vivo and in vivo conditions (for a review see [48]).

## Figures and Tables

**Table 1:** In vitro oxidation potential values (mV) of various electro active amino acids and peptides detected with DPV-µCFE in PBS at pH 7.4.

Substance Potential (mV)	
Tyrosin	720
Tryptophan	860
Cysteine	870
Neurotensin	670
Oxytocin	585
Vasopressin	610
Caerulein	670
Leu-enkephalin	605
Met-enkephalin	570
ACTH 1-24	650
ACTH 17-39	700
ß-endorphin	800
Somatostatin	805
Cholecystokinin (CCK-4)	730
Cholecystokinin (CCK-8)	810
LH-RH	700
Alpha-MSH	795

**Table 2:** In vivo effect of various treatments on DPV-µCFE striatal Peak 5 of anaesthetized rats. Data are presented as % of control (pretreatment) values, mean ± SD, * *p* < 0.05, Tukey test.

**LOCAL**	**TIME (min)**			
TREATMENT	0	40	80	120
NaCl 0.9%	100	102	95	93
2 µL, n = 9	±5	±8	±11	±16
SRIF	100	475 *	155 *	89
2 µg, n = 7	±9	±98	±41	±22
(C.A.)	100	122	136	114
2 µL, n = 7	±8	±18	±23	±11
(S.A.)	100	125	65 *	13 *
2 µL, n = 7	±5	±13	±11	±6
Bacitracin	100	130	159 *	153 *
10 ng, n = 5	±4	±13	±16	±14
GH	100	141 *	114	106
2 µg, n = 5	±6	±8	±7	±12
**SYSTEMIC**	**TIME (min)**			
TREATMENT	0	10	20	30
Cysteamine	100	65 *	22 *	8 *
100 mg/kg, n = 7	±10	±13	±11	
